# Repurposing of the Antipsychotic Trifluoperazine Induces SLC7A11/GPX4- Mediated Ferroptosis of Oral Cancer via the ROS/Autophagy Pathway

**DOI:** 10.7150/ijbs.99859

**Published:** 2024-11-11

**Authors:** Sheng-Chieh Tsai, Po-Chih Chang, Yu Tong Lin, Po-Tsang Huang, Jeff Yi-Fu Chen, Chang‑Shen Lin, Bin-Nan Wu, Hui-Min Chang, Wan-Ju Wu, Chi-I Chang, Chien-Hsing Lee

**Affiliations:** 1 Division of Pharmacology and Traditional Chinese Medicine, Graduate Institute of Medicine, College of Medicine, Kaohsiung Medical University, Kaohsiung City 80708, Taiwan.; 2 School of Medicine, College of Medicine, National Sun Yat-sen University, Kaohsiung City 804201, Taiwan.; 3 Division of Thoracic Surgery, Department of Surgery, Kaohsiung Medical University Hospital/Kaohsiung Medical University, Taiwan.; 4 Ph. D. Program in Biomedical Engineering, College of Medicine, Kaohsiung Medical University; Department of Sports Medicine, College of Medicine, Kaohsiung Medical University, Kaohsiung City 80708, Taiwan.; 5 AnTaimmu BioMed Co., Hsinchu City 300091, Taiwan.; 6 Department of Biotechnology, Kaohsiung Medical University, Kaohsiung City 80708, Taiwan.; 7 Department of Medical Research, Kaohsiung Medical University Hospital, Kaohsiung City 80708, Taiwan.; 8 Graduate Institute of Medicine, College of Medicine, Kaohsiung Medical University, Kaohsiung 80708, Taiwan.; 9 Department of Biological Science and Technology, National Pingtung University of Science and Technology, Pingtung City 91201, Taiwan.; 10 Research Centre for Active Natural Products Development, National Pingtung University of Science and Technology, Pingtung City 91201, Taiwan.; 11 Department of Pharmacology, School of Post-Baccalaureate Medicine, College of Medicine, Kaohsiung Medical University, Kaohsiung City 80708, Taiwan.; 12 Drug Development and Value Creation Research Center, Kaohsiung Medical University, Kaohsiung City 80708, Taiwan.

**Keywords:** Trifluoperazine, Oral Cancer, Ferroptosis, Autophagy, ROS, preventable death, mental disorder

## Abstract

Ferroptosis, a mode of cell death characterized by iron-dependent phospholipid peroxidation, has a substantial therapeutic potential for the treatment of various cancers. This study investigated the effects of trifluoperazine (TFP), an FDA-approved drug traditionally utilized for mental health disorder, on oral cancer cells, with a particular focus on the mechanisms involved in its potential anti-tumor properties. Our findings indicate that TFP significantly elevates the levels of lipid-derived reactive oxygen species (ROS) and induces ferroptotic cell death in oral cancer cells through pathways involving autophagy, the SLC7A11/GPX4 axis, and mitochondrial damage. Additionally, molecular docking analyses revealed that TFP acts as an inhibitor of GPX4. The elevated expression level of GPX4 in oral cancer biopsies was also found to correlate with a poor prognosis. Together, these results provide evidence that TFP selectively induces GPX4-mediated, autophagy-dependent ferroptosis, thereby exerting anti-cancer effects against oral cancer and preventable death.

## Introduction

Oral squamous cell carcinoma (OSCC), a prevalent malignancy that ranks as the sixth most commonly diagnosed cancer on a global scale, has a low survival rate and an unfavorable prognosis 1, 2. OSCC predominantly manifests within the tongue and gingiva, which represent the most frequent sites of occurrence 3, 4. Presently, therapeutic modalities include surgical resection, radiotherapy, and chemotherapy for OSCC management. However, the emergence of therapeutic resistance poses a significant challenge, leading to suboptimal treatment outcomes for a considerable proportion of OSCC patients 5. Consequently, the development of innovative chemotherapeutic agents is imperative to address the needs of individuals with advanced oral cancer.

Ferroptosis is a regulated form of cell death characterized by the iron-dependent accumulation of lethal reactive oxygen species (ROS) derived from lipids, particularly lipid hydroperoxides 6. Compounds that induce ferroptosis and possess anti-cancer potentials can be classified based on their mechanisms of action, including inhibition of the cysteine/glutamate antiporter (system X_c_^-^), direct interaction with glutathione peroxidase 4 (GPX4) and induction of iron oxidation and lipid peroxidation 7. Among those mechanisms, GPX4, a critical enzyme involved in cellular defense against oxidative damage, represents a promising target for eliminating cancer cells through ferroptosis 8. In addition, autophagy is an important cellular process that maintains homeostasis through self-degradation. According to recent research 9-11, autophagy plays an important role in the regulation of ferroptosis. The discovery of autophagy-ferroptosis interactions provides novel insights into cellular death and presents a significant therapeutic potential for cancer treatment.

**Figure 1:**
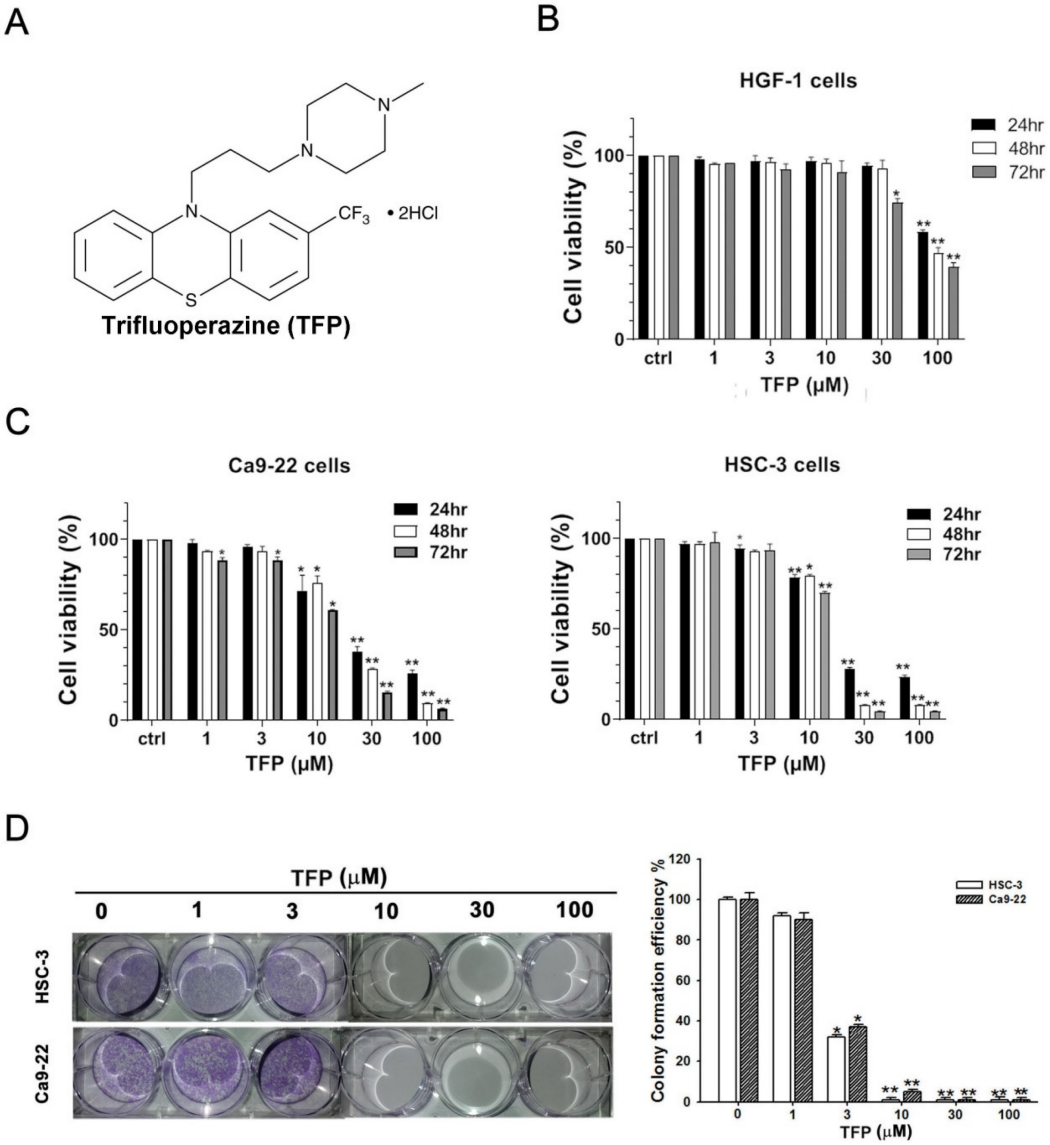

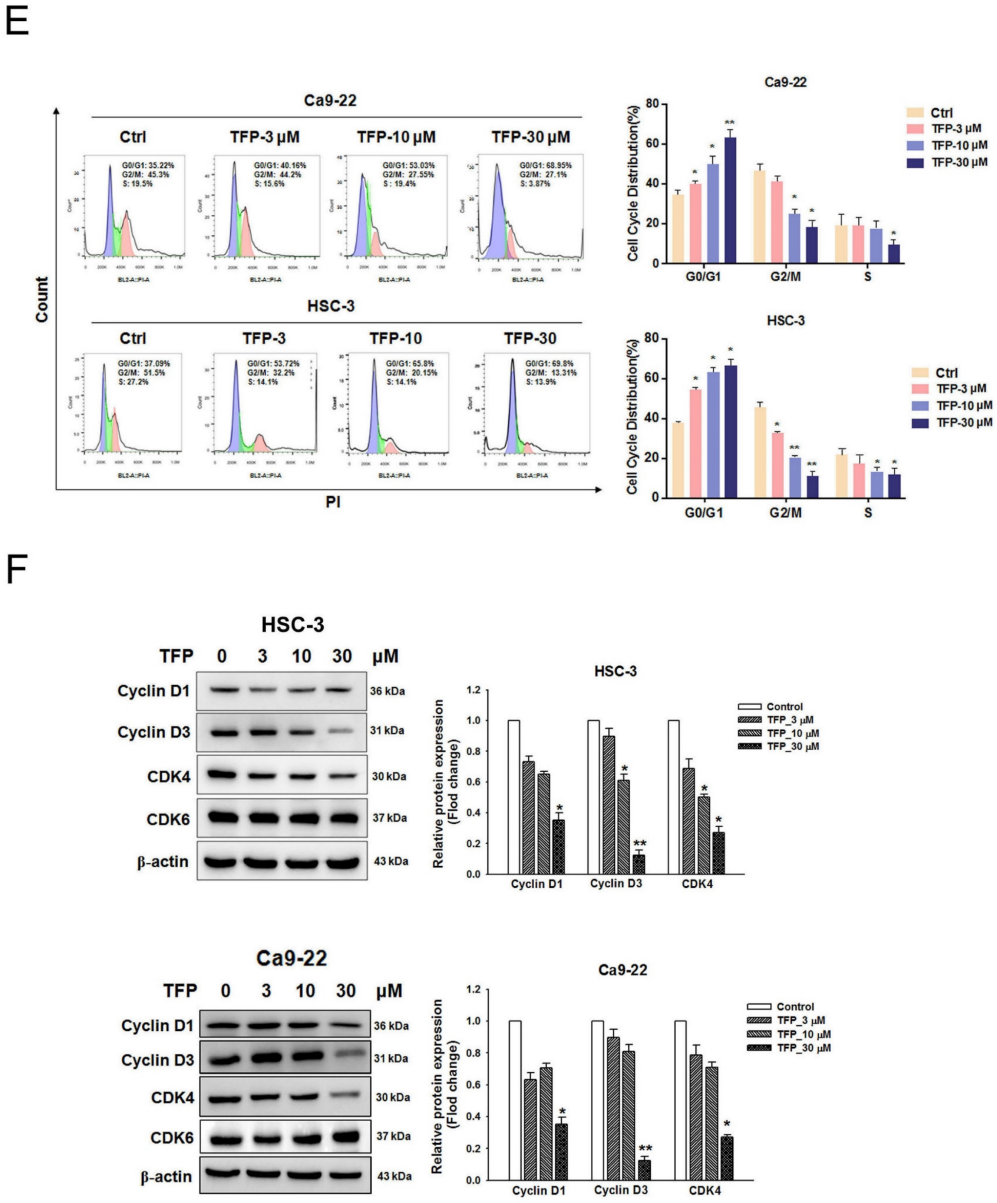
** TFP induces cell death, inhibits proliferation, and causes G0/G1 phase arrest in human oral cancer cells. (A)** Chemical structure of TFP. **(B)** Cell viability of the normal gingival fibroblast line HGF-1, and **(C)** OSCC cells (Ca9-22 and HSC-3) treated with various concentrations of TFP at different time points (24 h, 48 h and 72 h), was measured using the MTT assay. **(D)** Colony formation assays were conducted on OSCC cells (Ca9-22 and HSC-3) with or without TFP treatment. **(E)** The cell cycle distribution in TFP-treated OSCC cells (Ca9-22 and HSC-3) was analyzed using flow cytometry. **(F)** Western blot analysis was performed to examine the expression levels of cell cycle-related proteins, with β-actin serving as the internal control. Histograms present the statistical analysis of the relative expression levels of these proteins. Data are reported as means ± SD (n=3). Statistical significance is indicated as *P < 0.05, **P < 0.01 compared to the vehicle control group.

Trifluoperazine (TFP), an FDA-approved phenothiazine derivative (Fig.1A), is utilized for the treatment of schizophrenia and other psychiatric disorders. Extensive research has demonstrated the anti-proliferative properties of TFP against tumors 12-15 but the specific inhibitory mechanism of TFP on OSCC had not been elucidated. To characterize that, we used *in vitro* experiments and an *in vivo* zebrafish xenograft model to evaluate the suppressive effects of TFP on OSCC. Our study reveals that TFP induces autophagy-mediated ferroptosis in OSCC through the solute carrier family 7 member 11 (SLC7A11)/GPX4 pathway. These findings emphasize the potential therapeutic value of TFP for the treatment of OSCC.

## Materials and Methods

### Chemicals and reagents

DMEM medium and F12 medium were obtained from Gibco BRL (Gaithersburg, MD, USA). Fetal bovine serum (FBS), trypan blue, phosphate-buffered saline (PBS), dimethyl sulfoxide (DMSO), ribonuclease A (RNase A), acetic acid, methanol, carbobenzoxy-valyl-alanyl-aspartyl-[O-methyl]-fluoromethylketone (Z-VAD-FMK), 3-methyladenine (3-MA), 3-[4,5-dimethyl-thiazol-2-yl]-2,5-diphenyltetrazolium bromide (MTT), 5,5',6,6'-tetrachloro-1,1',3,3'-tetraethylbenzimidazolylcarbocyanine iodide (JC-1) and rapamycin were purchased from Sigma-Aldrich (St. Louis, MO, USA). The propidium iodide (PI)-FITC Annexin V apoptosis detection kit was purchased from BD Biosciences (San Jose, CA, USA). Antibodies against Cdk-4 (sc-23896), Cdk-6 (sc-7961), cyclin D1 (sc-8396), cyclin D3 (sc-245) and β-actin (sc-47778) were obtained from Santa Cruz Biotechnology (Santa Cruz, CA, USA). Antibodies against cleaved caspase-3 (#9661), PARP (#9542) and LC3B (#12741) were purchased from Cell Signaling (San Jose, CA, USA). Antibodies against caspase-9 (10380-AP-1) were purchased from Proteintech (Rosemont, IL, USA). All primary antibodies were diluted 1:1000. Anti-mouse and anti-rabbit IgG peroxidase-conjugated secondary antibodies (H+L) were purchased from Jackson ImmunoResearch (West Grove, PA, USA; 115-035-003 and 111-035-003) and diluted 1:200,000.

### Cell lines and culture

Ca9-22 (gingival carcinoma) and HSC-3 (tongue carcinoma) OSCC cell lines were obtained from the Health Science Research Resources Bank (HSRRB, Osaka, Japan). The normal human gingival fibroblast cell line (HGF-1) was obtained from the ATCC (Manassas, VA, USA). Cells were maintained in DMEM:F-12/3:2 in a humidified atmosphere at 37°C with 5% CO_2_ and were supplemented with 10% FBS, 2 mM glutamine, and antibiotics (normocinTM; InvivoGen, San Diego, CA, USA).

### Cell viability

Using the MTT assay according to the manufacturer's instructions, cell viability *in vitro* was detected. Normalized OD540 (EZ Read 400 Research, BioChrom, Holliston, MA, USA) values were used to calculate cell growth curves. Percentage changes in absorbance before and after different treatments were used to calculate percent cell viability.

### Colony formation assay

A density of 500 cells per well was plated in six-well plates for 24 h. The cells were treated with TFP, and the medium was replaced every two days. A 5% crystal violet stain was applied to live cells after 14 days, and colonies were counted using ImageJ software (NIH, MD, USA).

### Cell cycle assessment

To examine the effects of TFP on cell cycle distribution in human Ca9-22 and HSC-3 OSCC cells, staining with PI was performed as previously reported 16. In brief, 5 × 10^5^ cells were seeded into 10-cm petri dishes and treated with or without 10 or 30 µM TFP for 16 h. Subsequently, cells were harvested and stained with a PI staining kit according to the manufacturer's manual (BD Biosciences, San Jose, CA, USA). Cells were analyzed by flow cytometry utilizing a FACS CytoFlex S (Beckman, USA) and Flow Jo software (V.10.4, FlowJo, USA) was employed for data analysis.

### Assessment of intracellular ROS

Cells were treated with TFP for 24 h. DCFH-DA (2',7'-dichlorofluorescein diacetate) was diluted in serum-free culture medium at a 1:1000 ratio to obtain a 10 μM working solution. The cell culture medium was removed, and the DCFH-DA working solution was added in a volume sufficient to cover the cells. Cells were then incubated at 37°C for 20 min. Thereafter, the cells were washed three times with serum-free culture medium to remove any extracellular DCFH-DA that had not entered the cells. The intracellular ROS levels were subsequently analyzed using fluorescence microscopy (Leica, Munich, Germany) or flow cytometry (FACS CytoFlex S, Beckman, USA).

### Quantification of mitochondrial superoxide production

Mitochondrial superoxide levels were determined using the MitoSox Red reagent (Thermo Fisher, Sunnyvale, CA, USA). Following the appropriate treatments, the OSCC cells were washed three times with serum-free medium and then incubated with a 4 μM MitoSox Red staining solution at 37°C for 30 min. The red fluorescence of MitoSox within the cells was evaluated using fluorescence microscopy (Leica, Munich, Germany).

### Assessment of apoptotic nuclear changes

Human Ca9-22 and HSC-3 OSCC cells were seeded in 6-well plates and then treated as described. Following the treatments, the cells were stained with Hoechst 33342 dye (10 μg/mL) for 10 min. The stained cells were then observed using a fluorescence microscope (Leica, Munich, Germany) to evaluate the presence of apoptotic nuclear condensation.

### Assessment of Mitochondrial Membrane Potential (MMP)

MMP was evaluated using the JC-1 dye, following the manufacturer's instructions (Sigma-Aldrich, St. Louis, MO, USA). Cells were seeded in 6-well plates and then treated with varying concentrations of TFP for 24 h. After the treatment, the cells were incubated with JC-1 dye (1 μg/mL) for 10 min in the dark. Following the incubation, the cells were washed, resuspended in PBS, and analyzed using a FACS CytoFlex S (Beckman, USA). The ratio of red to green fluorescence, measured at excitation and emission wavelengths of 590 nm and 527 nm, respectively, was used to estimate the MMP.

### Measurement of mitochondrial respiration

The oxygen consumption rate (OCR) was quantified by following an established protocol 17. Mitochondrial respiration was evaluated using the Seahorse XF HS mini platform, an extracellular flux analyzer manufactured by Agilent Technologies (Santa Clara, CA, USA). Cells were seeded at a density of approximately 7,000 cells per well, and changes in cellular respiration were monitored continuously. To assess alterations in OCR, the cells were sequentially exposed to the following compounds: oligomycin, FCCP, and antimycin A/rotenone.

### Apoptosis assessment

An Annexin V-FITC/PI apoptosis detection kit from BD Biosciences (San Jose, CA, USA) was used to quantify apoptosis. Cells were seeded in 10-cm petri dishes (5×10^5^ cells/dish) and treated with TFP for 24 h. The cells were then harvested and then stained with PI and Annexin V-FITC in the dark for 15 min. Fluorescence of FITC and PI was detected using a FACS CytoFlex S (Beckman, USA). Cells that were early apoptotic (Annexin V-positive only) or late apoptotic (Annexin V- and PI-positive) were measured and analyzed using FlowJo software (Tree Star Inc., San Carlos, CA, USA).

### Measurement of cleaved caspase-3

Caspase-3 is considered to be an important biomarker of apoptosis. The measurement of caspase-3 was performed using a human cleaved caspase-3 ELISA kit (Abcam #Ab220655, Paris, France) according to the manufacturer's protocol.

### Detection of Acidic Vesicular Organelles (AVOs)

Human OSCC cells (1 × 10^5^) were seeded onto coverslips and allowed to adhere. Following treatment with DMSO (control) and the specified concentrations of TFP for 24 h, the cells were stained with 1 μg/mL AO for 15 min, washed with PBS, and imaged using a Leica fluorescence microscope at a ×100 objective lens magnification. The acidity, autophagic lysosomes appeared as orange/red fluorescent cytoplasmic vesicles, whereas the nuclei were stained green. The percentage of AVOs (exhibiting characteristic red fluorescence) was quantified by analyzing at least 100 cells per image using a fluorescence microscope (Leica, Munich, Germany) for each experimental condition.

### Western blot analysis

Cells were harvested and lysed, after which the lysates were centrifuged to obtain protein-containing supernatants. The protein concentrations were determined, and equal amounts were separated by SDS-PAGE and then electrotransferred onto membranes. Each membrane was blocked with 5% non-fat milk and incubated with primary and secondary antibodies. Signals were detected using an ECL kit (Amersham, Buckinghamshire, UK), and protein bands were visualized using a Chemiluminescence Imaging System (Vilber-Vilber Fusion Fx6 Edge, Marne La Vallee, France).

### Iron assay

The intracellular ferrous iron (Fe^2+^) content was quantified using an Iron Assay Kit (ab83366, Abcam, Paris, France). Cells were harvested after treatment with TFP for 24 h, and then homogenized in assay buffer on ice. Homogenates were centrifuged at 13,000 × g for 10 min at 4°C, and 300 μL supernatant was collected from each sample. Iron reducer (300 μL) was added, mixed, and incubated at room temperature for 30 min. Iron probes (200 μL) were then added, mixed, and incubated for 30 min at ambient temperature, protected from light. Absorbance was measured at 593 nm using a colorimetric plate reader.

### Measurement of malondialdehyde (MDA)

Cellular MDA levels were quantified using an MDA assay kit (S0131, Beyotime, Shanghai, China), following the manufacturer's instructions. A total of 3 × 10^6^ cells were harvested after treatment with the indicated concentration of TFP.

### Lipid peroxidation analysis

A total of 2 × 10^5^ cells were incubated with 5 μM C11-BODIPY^581/591^ (D3861, Invitrogen, USA) for 30 min. Subsequently, the cells were washed three times with PBS, resuspended in PBS, and analyzed using an FACS CytoFlex S (Beckman, USA). Flow Jo software (V.10.4, FlowJo, USA) was employed for data analysis.

### Zebrafish xenograft assay

Zebrafish (Danio rerio) were obtained from the Taiwan Zebrafish Core Facility at Academia Sinica. Zebrafish care and maintenance complied with animal regulations at Kaohsiung Medical University. Zebrafish were kept at 28.5°C with a 14/10 h light/dark cycle. The zebrafish xenograft assay was used to confirm the inhibitory effect of TFP on OSCC cell proliferation, in accordance with the 3Rs principles and IACUC approval (IACUC Approval No. KMU-IACUC-111122). HSC-3 OSCC cells were labeled with DiI dye and were then microinjected into the yolk sac of embryos (200 cells/embryo) at 72 h post-fertilization (hpf) for analysis using fluorescence microscopy. The embryos (n = 30/group) were treated with or without TFP at the indicated h post-injection (hpi). The zebrafish model enables tracking of labeled cancer cells and evaluation of the anti-proliferative activity of TFP, with the procedures following a previously published protocol with minor modifications 18.

### The Cancer Genome Atlas (TCGA) dataset

The analysis file contains expression subtypes data from the previous study 19. A total of 279 patients are included in this file. The oral site is examined anatomically, including the alveolar ridge, base of the tongue, buccal mucosa, floor of the mouth, hard palate, lip, oral cavity, and oral tongue. Kaplan-Meier survival analysis is performed using data obtained from http://kmplot.com/analysis/.

### Structure preparations for GPX4

The crystal structure of apo (ligand-free) wild-type human GPX4 was determined at 1.0 Å resolution (PDB ID: 6HN3). The overall fold and active site are conserved compared to previous mutant structures. A mass spectrometry-based approach, combined with a surface mutation, enabled determination of the GPX4-covalent inhibitor ML162 complex structure. This method provides opportunities to obtain co-complex structures of GPX4, a potential cancer drug target, with various inhibitors, offering valuable insights into binding interactions and inhibition mechanisms to aid in the design of potent and selective GPX4 inhibitors.

### Molecular docking and docking pose analysis

The crystal structure of human GPX4 (PDB ID: 6HN3) was protonated using the Amber10:EHT force field. Putative ligand-binding pockets were identified on the protein surface. Multiple conformations of a test compound were docked into these pockets using an induced-fit protocol. The Triangle Matcher algorithm generated initial poses, London ΔG ranked them, and GBVI/WSA ΔG scored the final optimized poses. The docking poses per pocket were analyzed. This computational framework evaluated potential small-molecule binding sites and interactions with GPX4, providing insights to guide the design of selective GPX4 inhibitors as therapeutic candidates. All analyses were performed using Molecular Operating Environment (MOE) software (Chemical Computing Group, Montreal, Quebec, Canada).

### Statistical analysis

Differences between TFP- and DMSO- (as vehicle control) treated cells were analyzed in the indicated number of independently performed experiments. Statistical analyses were carried out using GraphPad Prism 8 software package or SPSS 19.0 software (SPSS Inc., USA). Student's t-test and one-way ANOVA were used to analyze differences in the data. *P < 0.05, **P < 0.01 were considered to indicate statistical significance.

## Results

### TFP triggers cell death, inhibits cell proliferation, and promotes cell cycle arrest at G0/G1 in OSCC cells

HSC-3 and Ca9-22 cells, which are oral cancer cells, along with HGF-1 cells, which are normal oral cells, were treated with TFP for 24, 48, and 72 h. MTT assays demonstrated a significant decrease in cell viability of HSC-3 and Ca9-22 oral cancer cells due to TFP treatment (Fig. 1C). After 24 h of treatment, HSC-3 and Ca9-22 cells exhibited IC_50_ values of 26.65 ± 1.1 µM and 23.49 ± 1.26 µM, respectively. TFP had much less of a toxic effect on HGF-1 normal oral cells at the indicated concentrations (Fig. 1B). TFP treatment resulted in a substantial reduction in the number of colonies formed by OSCC cells (Fig. 1D). These findings indicate that TFP inhibits the proliferation of OSCC cells in a dose- and time-dependent manner.

The abnormal cell cycle progression of cancer cells leads to uncontrolled proliferation and tumor formation 20. To explore the potential cause of the inhibition by TFP, flow cytometry analysis was conducted to examine if TFP treatment led to cell cycle arrest. We observed a significant increase in the number of cells arrested in the G0/G1 phase following TFP treatment (Fig. 1E). To further elucidate the molecular basis of that phenomenon, the expression levels of cell cycle-related proteins were analyzed using Western blotting (Fig. 1F). TFP treatment resulted in decreased expression levels of cyclin D1, cyclin D3 and CDK4 in both OSCC cell lines. Taken together, the results showed that TFP, by initiating G0/G1-phase arrest, potently reduces cell proliferation and viability, and even causes cell death, in OSCC cells.

### TFP leads to ROS production and mitochondrial damage in OSCC cells

**Figure 2:**
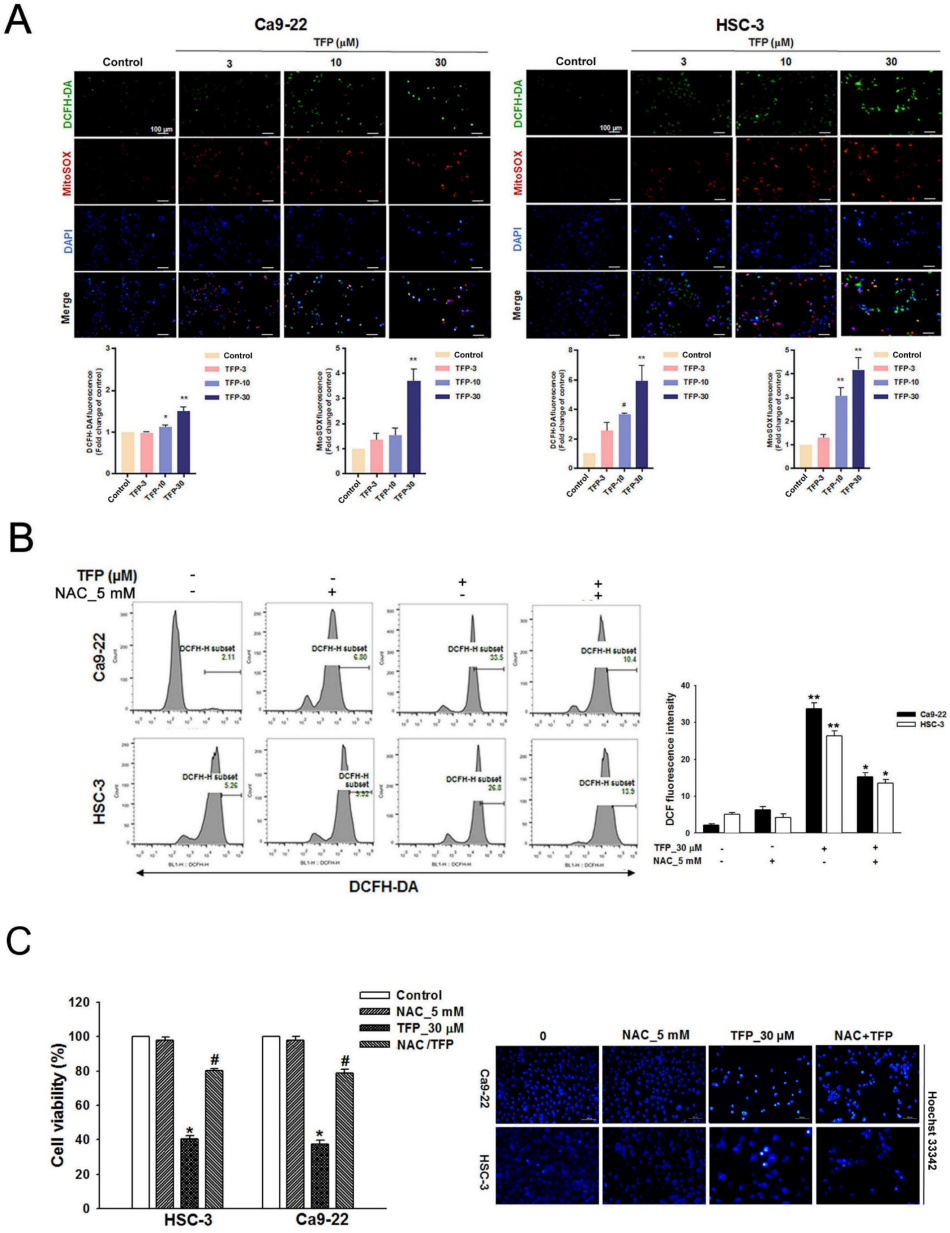

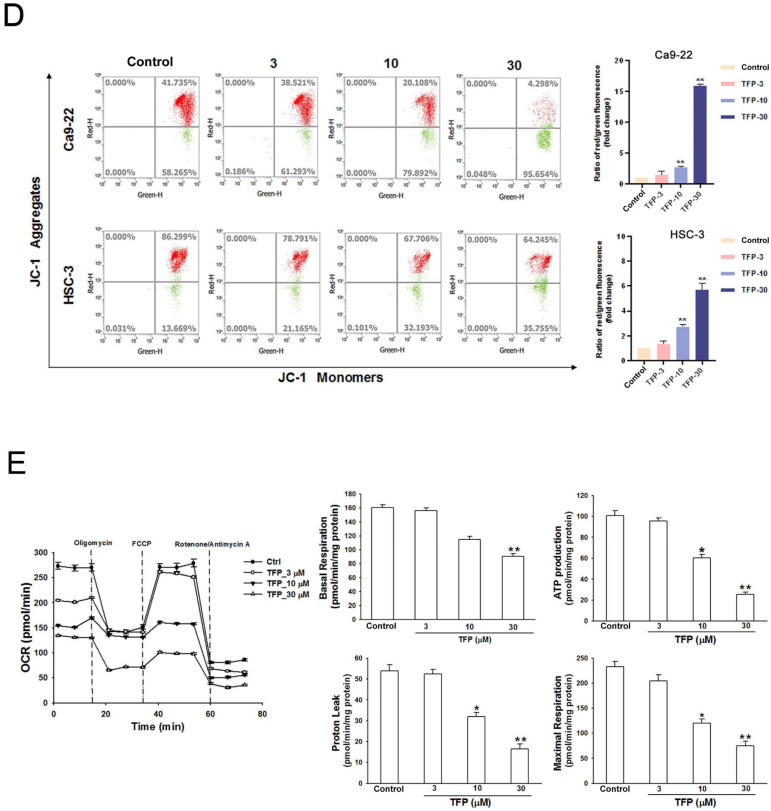
** TFP induces ROS production and mitochondrial damage in human oral cancer cells. (A)** OSCC cells (Ca9-22 and HSC-3) were treated with TFP (3, 10, 30 μM) or vehicle control. Cells were stained with DCFH-DA (2 μM), MitoSOX (2 μM), and DAPI (5 μM) for 24 h, and quantified using immunofluorescence assays. Scale bars = 100 μm. **(B)** OSCC cells (Ca9-22 and HSC-3) were pretreated with NAC (5 mM for 1 h), then treated with 30 μM TFP for 8 h after which the cells were analyzed by flow cytometry. **(C)** Cell viability and fluorescence microscopy images of OSCC cells (Ca9-22 and HSC-3) treated with TFP with or without NAC for 24 h and then stained with DAPI (1 μM). Scale bars = 100 μm. **(D)** OSCC cells (Ca9-22 and HSC-3) were treated with TFP (3, 10, 30 μM) or vehicle control. MMP was assessed using JC-1 staining and quantified via flow cytometry. **(E)** Oxygen consumption rate (OCR) curves were plotted for OSCC cells (Ca9-22 and HSC-3) treated with the indicated concentrations of TFP for 24 h. Cells were analyzed using a Seahorse Bioscience XF24 analyzer with or without oligomycin, FCCP, and rotenone/antimycin A to measure OCR (pMol/min/1×10⁴ cells). Quantification of parameter changes induced by TFP treatment is shown. Data are reported as means ± SD (n=3). Statistical significance is indicated as *P < 0.05, **P < 0.01 compared to the vehicle control group.

ROS can induce cell death, thus acting as a tumor suppressor 21. Flow cytometry showed that the fluorescence intensities of both DCFH-DA and MitoSOX Red increased with an increased concentration of TFP in both OSCC cell lines (Fig. 2A). TFP-induced ROS production was inhibited by the ROS inhibitor N-acetyl cysteine (NAC) (Fig. 2B). Furthermore, co-treatment with TFP and NAC effectively reduced TFP-induced cell death (Fig. 2C). In addition, MMP, an important parameter reflecting mitochondrial function, was assessed using JC-1 staining. Treatment with TFP resulted in a decrease in MMP in both OSCC cell lines, as indicated by increased green fluorescence (Fig. 2D), suggesting that TFP disrupts mitochondrial function.

To further investigate whether the metabolic reprogramming in OSCC cells that was inhibited by TFP treatment was related to mitochondrial function, we examined those parameters using a Seahorse XF cell mito stress test kit in HSC-3 oral cancer cells. Notably, when exposed to a TFP concentration of 30 μM, the parameters of respiration (including basal respiration, ATP production, maximum respiration, and proton leak) exhibited significant reductions (Fig. 2E). These findings suggest that TFP may adversely affect the mitochondrial network due to impaired respiration capacity. Taken together, these findings strongly suggest that TFP-induced cell death of OSCC cells is due to ROS-mediated mitochondrial dysfunction.

### TFP induces apoptotic, autophagic and ferroptotic cell death in OSCC cells

**Figure 3:**
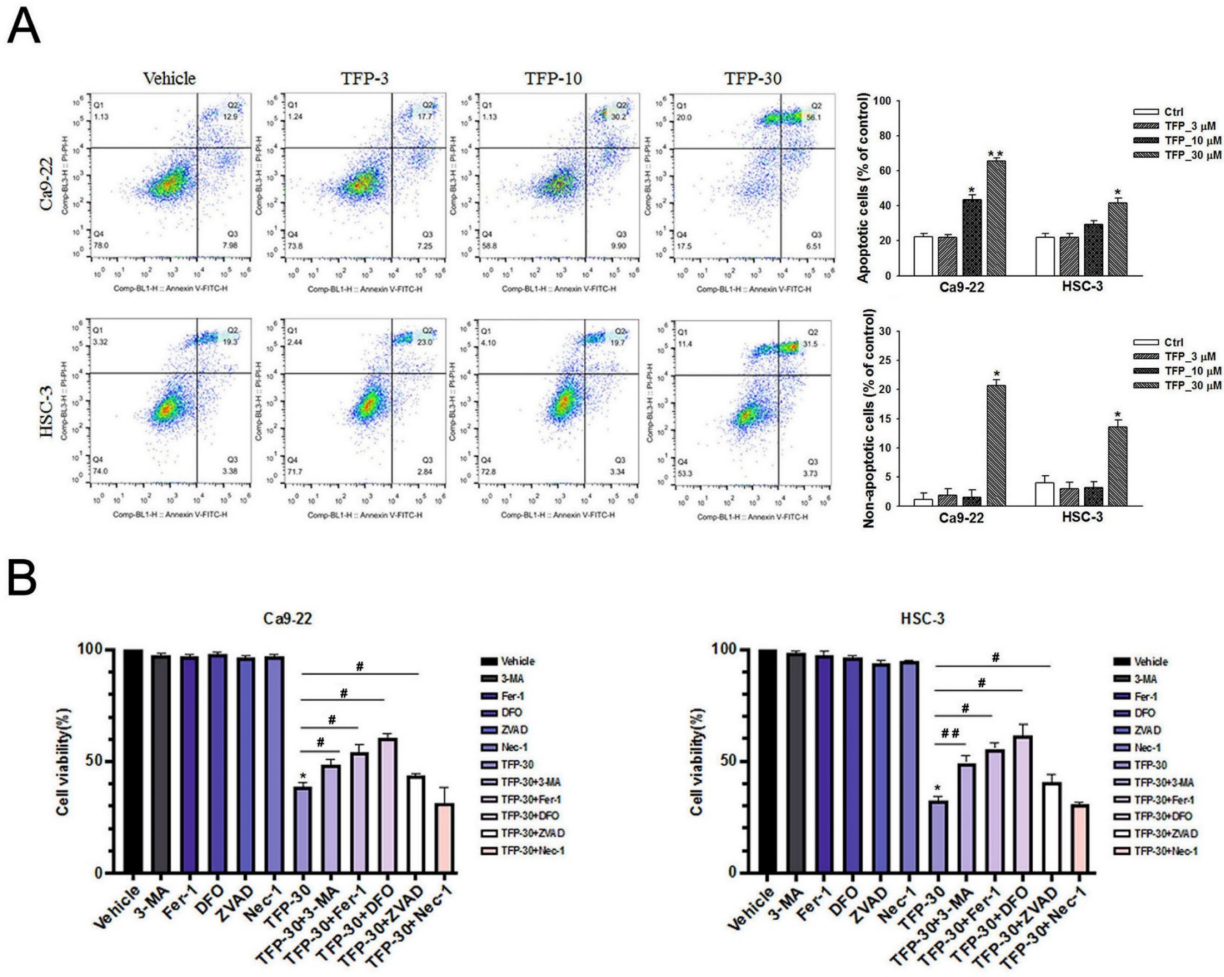

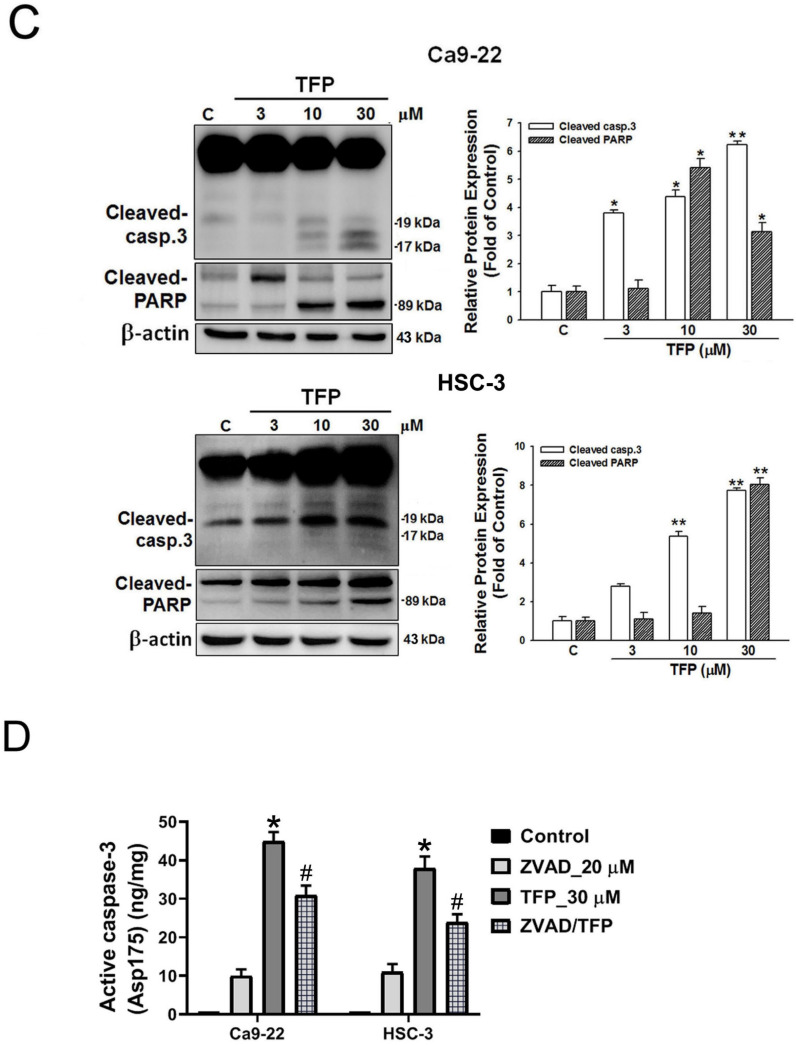
** TFP induces apoptotic, autophagic and ferroptotic cell death in human oral cancer cells. (A)** Analysis of apoptosis and non-apoptotic deaths in OSCC cells (Ca9-22 and HSC-3) after TFP treatment (3, 10 or 30 μM) for 24 h using Annexin V-FITC/PI staining with flow cytometry detection. Quantification analysis of the percentage of apoptotic and non-apoptotic death is shown. The upper left Annexin V-negative, PI-positive (Annexin V-, PI+) cells are defined as non-apoptotic cells. The upper right quadrant shows Annexin V-positive, PI-positive (Annexin V+, PI+) cells, which are defined as late apoptotic cells. The lower right quadrant shows Annexin V-positive, PI-negative (Annexin V+, PI-) cells, which are defined as early apoptotic cells. **(B)** OSCC cells (Ca9-22 and HSC-3) were treated with TFP with or without 3-MA (5 mM), Ferrostatin-1 (Fer-1, 5 µM), Deferoxamine (DFO, 10 µM), Necrostatin-1(Nec-1, 10 µM) or Z-VAD-FMK (ZVAD, 20 μM) for 24 h, after which cell viability was assessed using the MTT assay. **(C)** OSCC cells (Ca9-22 and HSC-3) were treated with TFP (3, 10 or 30 μM) for 24 h. The expression levels of apoptosis-associated proteins were determined by western blot. β-actin was used as an endogenous reference. Histograms represent the statistical analysis of the relative expression level of apoptosis-associated proteins. **(D)** Cleaved caspase 3 activity was analysed in TFP-treated OSCC cells (Ca9-22 and HSC-3) with or without ZVAD using ELISA. Data are reported as means ± SD (n=3). ^*^P < 0.05, ^**^P < 0.01 compared to the vehicle control group. ^#^P < 0.05, ^##^P < 0.01 compared to the TFP-treated group.

TFP has the ability to suppress cell viability and induce cell death, so we characterized the type of cell death caused by TFP. Annexin V-FITC/PI staining and flow cytometry analysis was used to investigate the impact of TFP on OSCC cells. Our results indicate that treatment with TFP led to a dose-dependent increase in the proportion of early (Annexin V+, PI-) and late (Annexin V+, PI+) apoptotic cells (Fig. 3A). Further, TFP was also found to induce non-apoptotic cell death (Annexin V-, PI+) (Fig. 3A). To investigate the regulatory mechanisms underlying these alternative cell death modes, inhibitors targeting the different pathways were employed, and the MTT assay was utilized for analysis. OSCC cells were treated with 30 µM TFP in combination with an autophagy inhibitor (3-MA at 5 mM), ferroptosis inhibitors (Ferrostatin-1; Fer-1 at 5 µM and Deferoxamine; DFO at 10 µM), an apoptosis inhibitor (Z-VAD-FMK; ZVAD at 20 µM), and a necrosis inhibitor (Necrostatin-1 at 50 µM). The results (Fig. 3B) indicate that the apoptotic, autophagic and ferroptotic inhibitors significantly enhanced the reversal of TFP-induced effects compared to the other cell death inhibitors. Therefore, these findings suggest that TFP can regulate the survival of OSCC cells through the mechanisms of apoptosis, autophagy and ferroptosis.

Apoptotic signaling cascades are initiated by caspases 22. Activated cytoplasmic endonucleases then cleave a wide range of substrates, including poly (ADP-ribose) polymerase (PARP), as a hallmark of apoptosis 23. Western blot analysis showed that TFP upregulated the levels of active caspase-3 and PARP cleavage protein (Fig. 3C). Treatment with ZVAD, a pan-caspase inhibitor, mitigated the TFP-induced cleaved caspase-3 activity (Fig. 3D). These results suggest that TFP-induced apoptosis in oral cancer cells may be facilitated by caspase activation mechanisms.

### TFP induces ROS-mediated autophagy in OSCC cells

**Figure 4:**
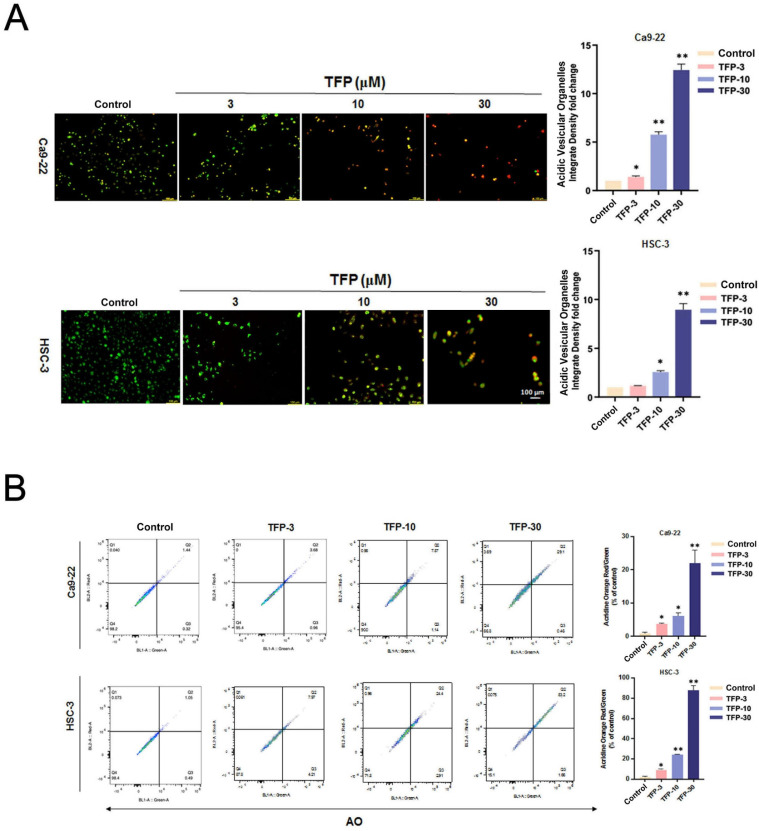

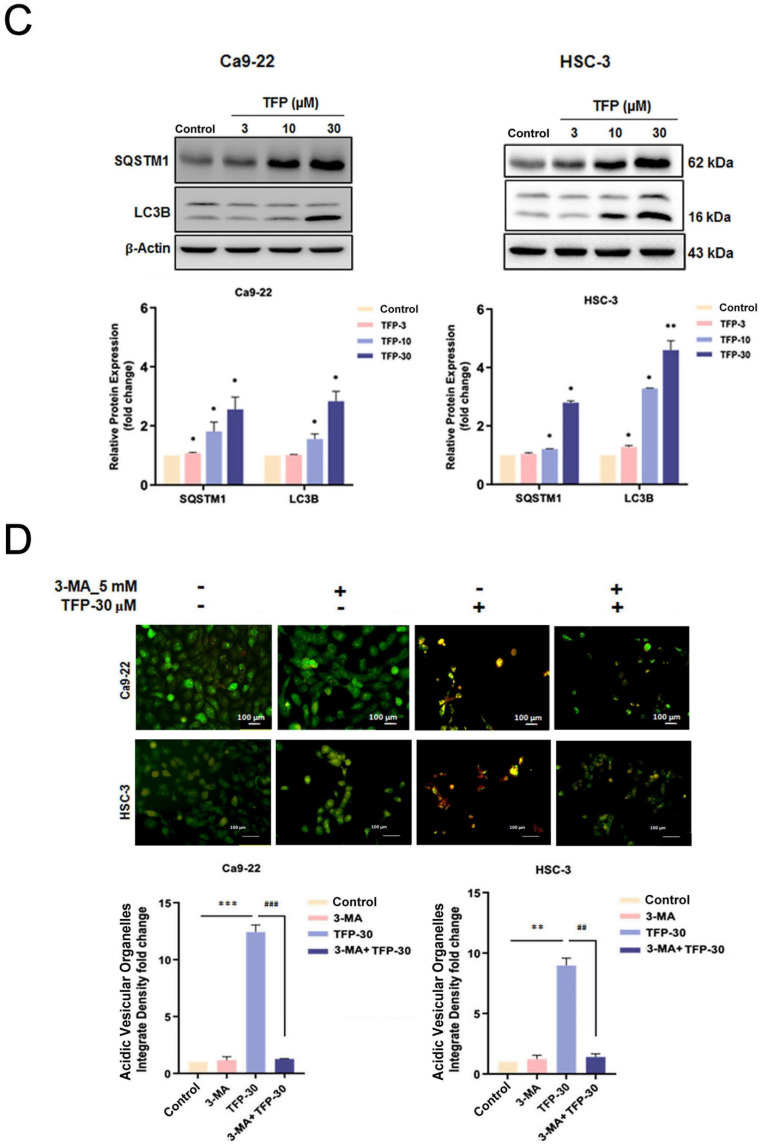

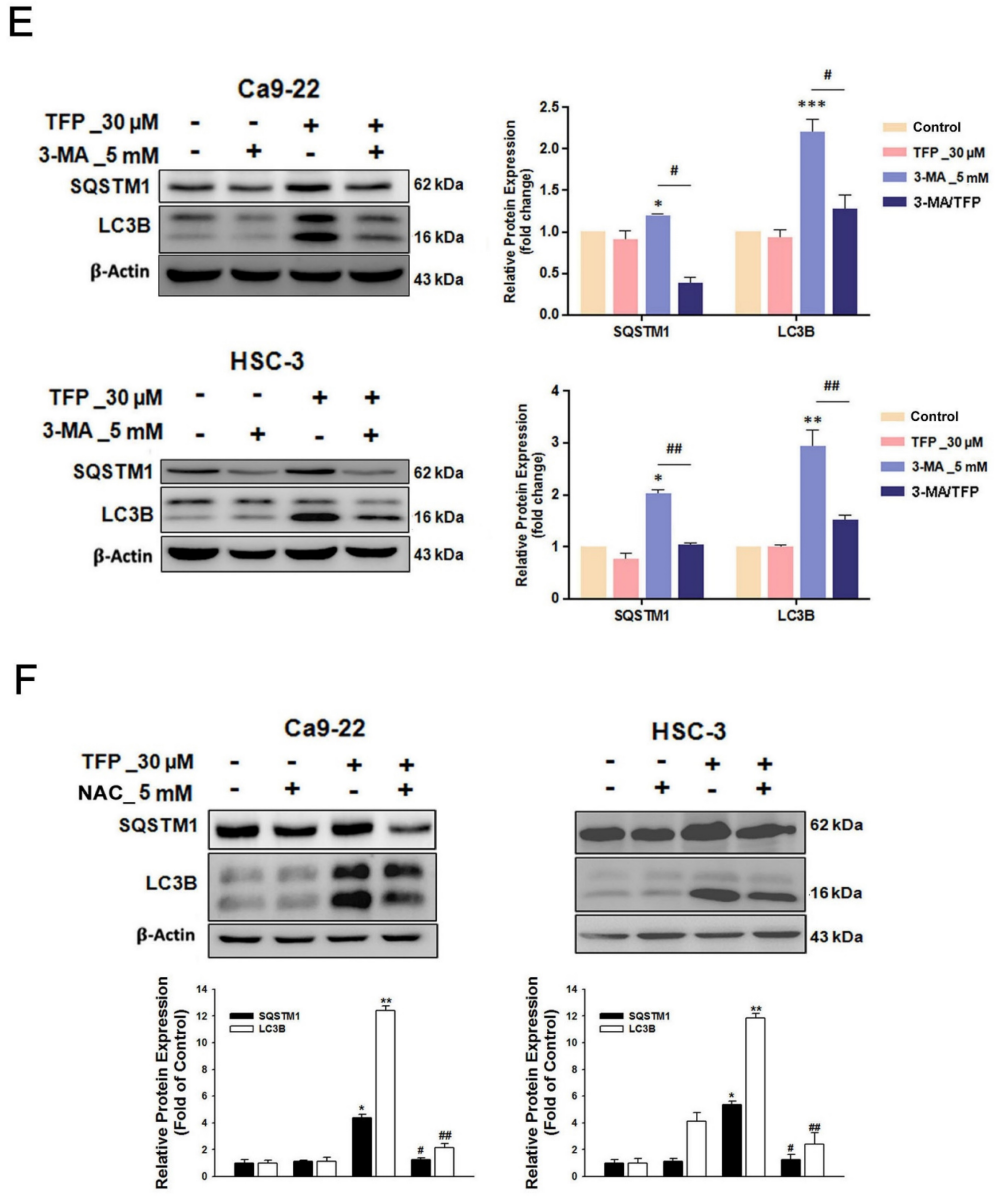
** TFP induces ROS-mediated autophagy in human oral cancer cells. (A)** Fluorescence microscopy using AO staining revealed the formation of acidic vesicular organelles (AVOs) in OSCC cells (Ca9-22 and HSC-3) following TFP treatment. The orange/red color indicates AVOs, whereas the nuclei were stained green. **(B)** Flow cytometry was employed to determine the mean red-to-green fluorescence ratio in AO-stained cells. **(C)** OSCC cells (Ca9-22 and HSC-3) were exposed to TFP (10 or 30 μM) for 24 h. The expression levels of autophagy-related proteins were assessed by western blot, with β-actin serving as the internal reference. The histograms illustrate the statistical analysis of the relative expression levels of these proteins. **(D)** OSCC cells (Ca9-22 and HSC-3) were treated with 30 μM TFP in the presence or absence of 3-MA (5 mM). Fluorescence microscopy of AO staining assays was performed to visualize AVOs. **(E)** Western blot analysis was conducted to measure the levels of autophagy-associated proteins, using β-actin as an endogenous control. **(F)** OSCC cells (Ca9-22 and HSC-3) were treated with 30 μM TFP with or without NAC (5 mM). The expression of autophagy-related proteins was analyzed via western blot, with β-actin as the internal reference. Data are reported as means ± SD (n=3). Statistical significance is indicated as follows: *P < 0.05, **P < 0.01, ***P < 0.001 compared to the vehicle control group; ^#^P < 0.05, ^##^P < 0.01, ^###^P < 0.001 compared to the TFP-treated group.

Autophagy plays a vital role in determining cellular survival, death, differentiation and development through the lysosomal degradation pathway 23. Acridine orange (AO) staining can be used to detect AVOs that are formed during autophagy 24. First, we examined the intracellular morphology of OSCC cells using AO staining to see whether autophagy was induced. TFP treatment significantly increased the level of AVO formation (orange/red fluorescent) in OSCC cells (Fig. 4A). In addition, flow cytometry analysis revealed a concentration-dependent increase in autophagic cells (Fig. 4B). TFP upregulated the expression of the autophagy-related proteins LC3B-II and SQSTM1 (Fig. 4C). Furthermore, pretreatment with 3-MA attenuated the TFP-induced increase in autophagic cells and the expression levels of LC3B-II and SQSTM1 (Fig. 4D, E). Expression levels of LC3B-II and SQSTM1 were also reduced during pretreatment with the antioxidant NAC (Fig. 4F). These findings suggest that modulation of ROS might be involved in the TFP-induced autophagy in OSCC cells.

### Ferroptosis is a determinant of TFP-induced cell death in OSCC cells

**Figure 5:**
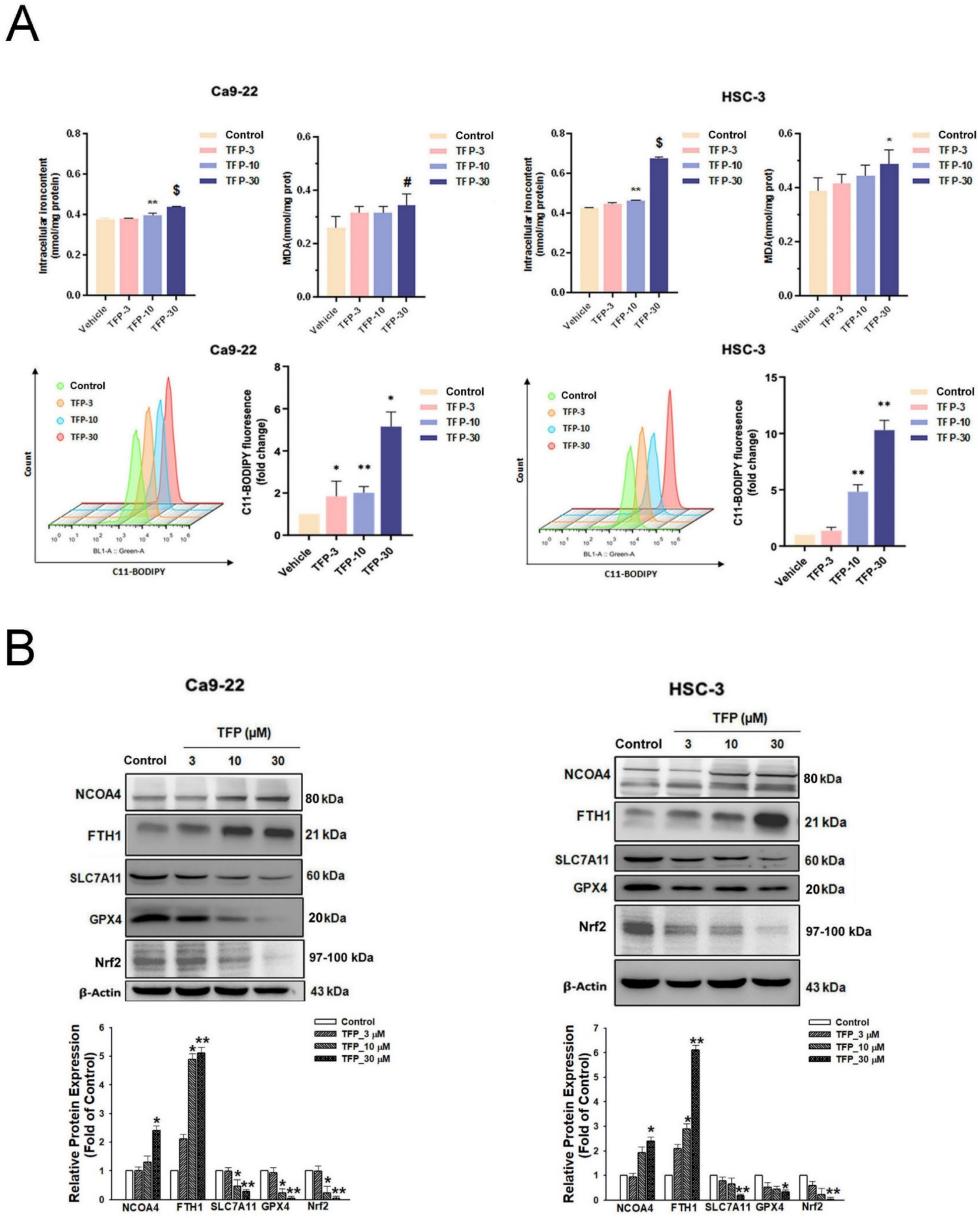

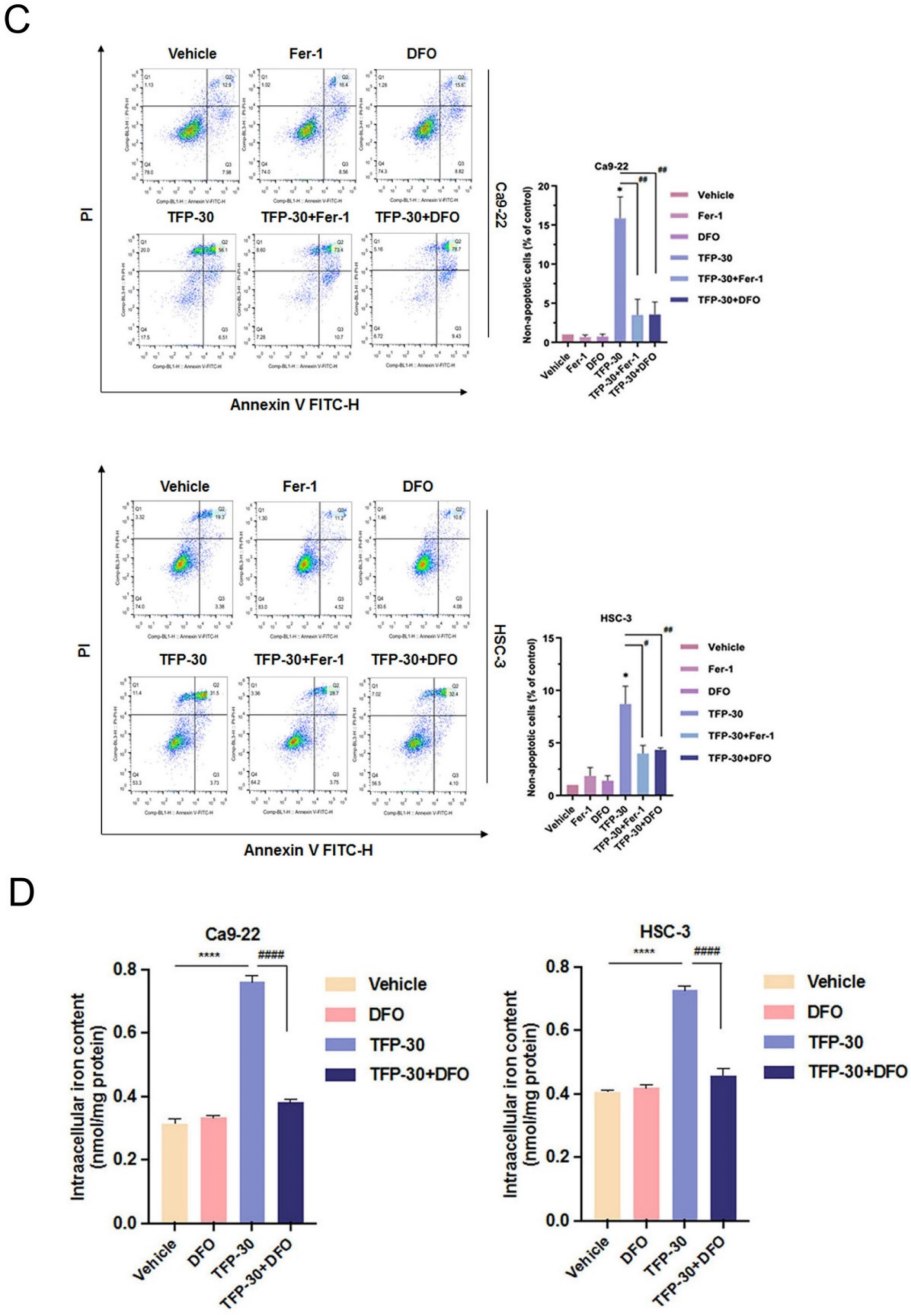

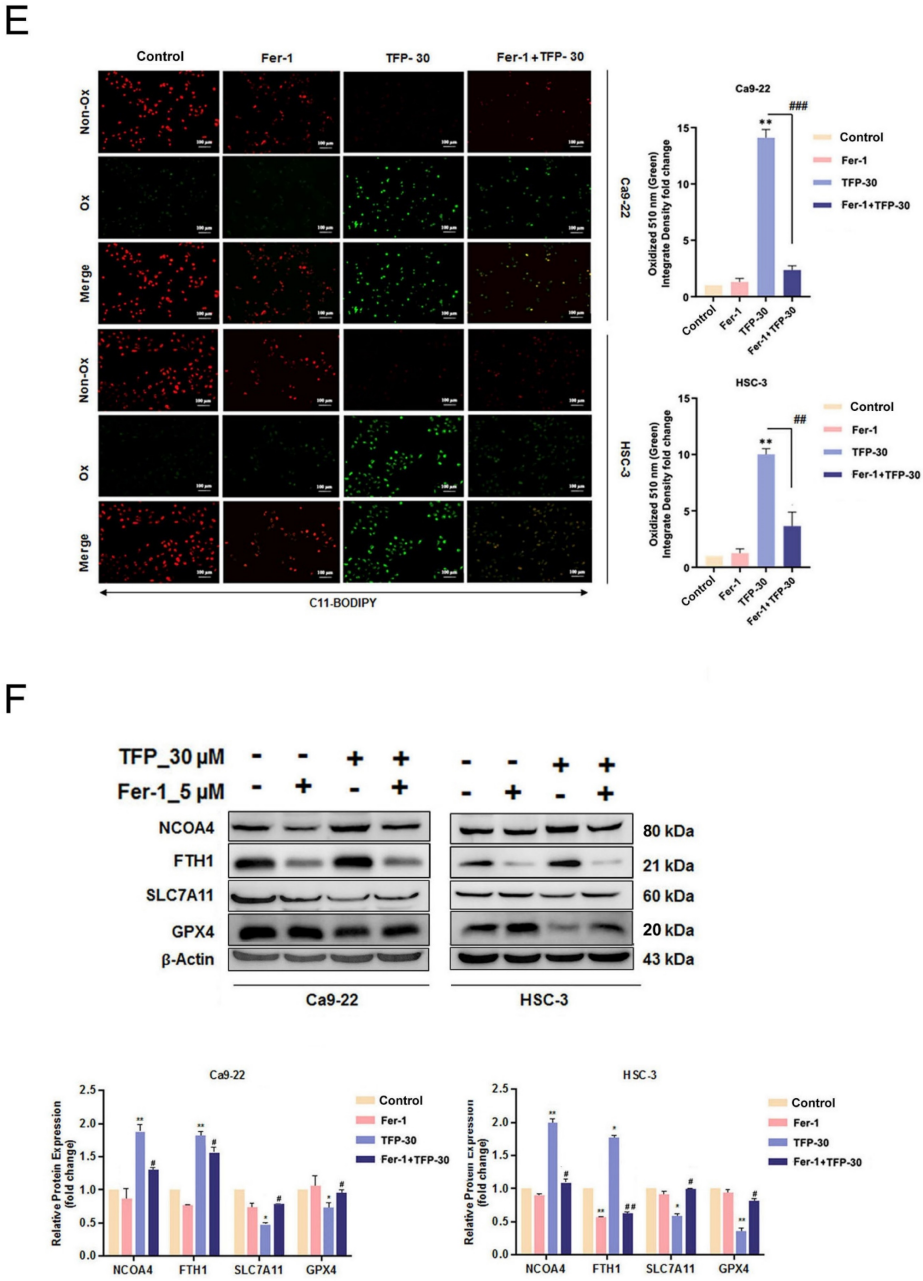
** TFP triggers ferroptosis in human oral cancer cells. (A)** Intracellular Fe^2+^ levels were measured in OSCC cells using an iron assay kit. Lipid peroxidation was detected using a MDA assay kit and C11-BODIPY^581/591^ fluorescent ratio-probe after 24 h treatment of TFP. **(B)** The expression of ferroptosis-related proteins analyzed by western blotting. β-actin was used as control. Histograms represent the statistical analysis of the relative expression level of ferroptosis-associated proteins. OSCC cells treated with 30 μM TFP in the absence or presence of ferroptosis inhibitors (Ferrostatin-1; Fer-1 at 5 µM and Deferoxamine; DFO at 10 µM). **(C)** Analysis of apoptosis death using Annexin V-FITC/PI staining with flow cytometry detection. Quantification analysis of the percentage of apoptotic death is shown. **(D)** Intracellular Fe^2+^ levels were measured using an iron assay kit. **(E)** Lipid peroxidation was detected using a C11 BODIPY 581/591 fluorescent ratio-probe. **(F)** The expression of ferroptosis-related proteins by western blotting. β-actin was used as a control. Histograms represent the statistical analysis of the relative expression level of ferroptosis-associated proteins. Data are reported as means ± SD (n=3). *P < 0.05; ***P < 0.01; ***P < 0.001 compared with the control, ^#^P < 0.05; ^##^P < 0.01,^ ###^P < 0.001 compared with the TFP-treated group.

The results of our study showed that TFP treatment increased intracellular iron levels and MDA levels, markers of lipid peroxidation in oral cancer cells (Fig. 5A). In addition, the data in Figure 5A show that TFP induced significant lipid ROS accumulation as indicated by the increase in oxidized C11-BODIPY^581/591^ probe in oral cancers, indicating the induction of ferroptosis in TFP-treated oral cancers. Furthermore, we examined the expression of several proteins closely associated with the ferroptosis process in oral cancer cells following TFP treatment. Treatment with TFP significantly upregulated the expression levels of pro-ferroptotic proteins, such as FTH1 and NCOA4, while downregulating the expression levels of proteins that reverse ferroptosis, such as SLC7A11, GPX4 and Nrf2, in oral cancer cells (Fig. 5B). The TFP-induced increase in non-apoptotic cells, intracellular iron levels, and lipid ROS were attenuated significantly by co-treatment with the ferroptosis inhibitors Fer-1 or DFO in oral cancer cells (Fig. 5C-E). Further analysis showed that Fer-1 pretreatment altered NCOA4, FTH1, SLC7A11 and GPX4 expression in TFP-treated cells (Fig. 5F). As a result of these findings, it seems that TFP causes a form of cancer in oral cancer cells by inducing ferroptosis via the modulation of key proteins involved in that process, which leads to an accumulation of iron-dependent lipid peroxides and subsequent cell death.

### TFP induces autophagy-mediated ferroptosis through ROS in OSCC cells

**Figure 6:**
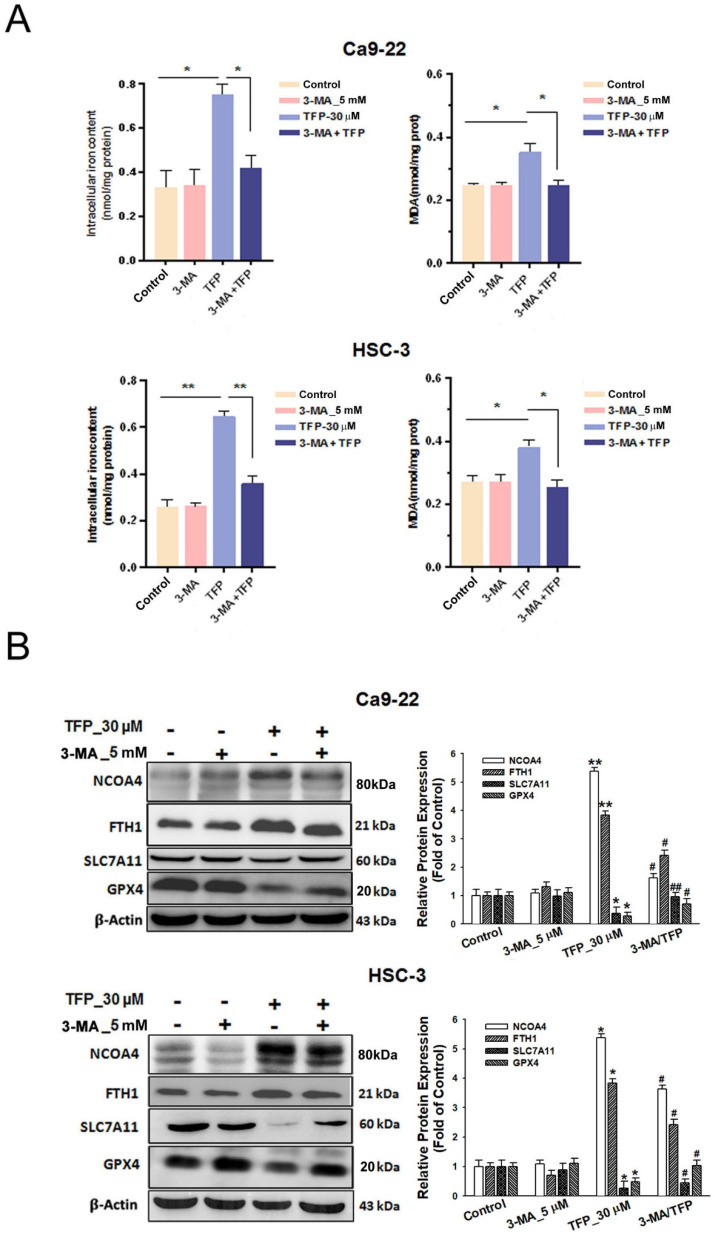

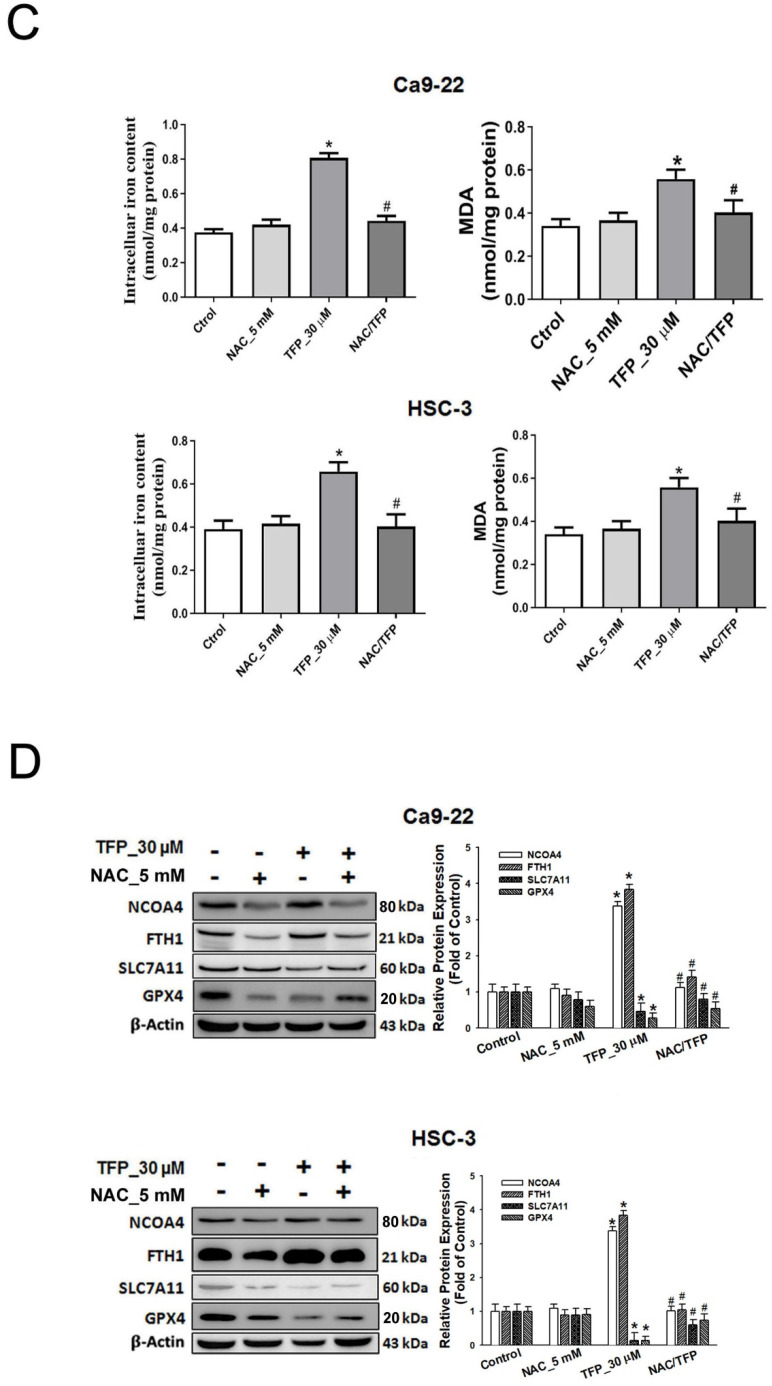
** TFP induces autophagy-mediated ferroptosis via ROS in human oral cancer cells. (A)** OSCC cells were treated with 30 μM TFP with or without 3-MA. Intracellular Fe²⁺ levels were quantified using an iron assay kit, and lipid peroxidation was assessed using an MDA assay kit. **(B)** Expression levels of ferroptosis-related proteins were analyzed by western blotting, with β-actin serving as the internal control. Histograms depict the statistical analysis of the relative expression levels of these proteins. **(C)** OSCC cells were treated with 30 μM TFP in the presence or absence of NAC. Intracellular Fe²⁺ levels were measured using an iron assay kit, and lipid peroxidation was detected using an MDA assay kit. **(D)** Western blot analysis was conducted to determine the expression levels of ferroptosis-related proteins, with β-actin used as the loading control. Histograms represent the statistical analysis of the relative expression levels of these proteins. Data are reported as means ± SD (n=3). Statistical significance is indicated as follows: *P < 0.05, **P < 0.01, ***P < 0.001 compared to the control; #P < 0.05, ##P < 0.01, ###P < 0.001 compared to the TFP-treated group.

Pretreatment with the autophagy inhibitor 3-MA attenuated the TFP-induced elevation in intracellular iron and MDA levels in oral cancer cells (Fig. 6A). The reduced expression of pro-ferroptotic proteins, NCOA4 and FTH1, as well as the increased expression of anti-ferroptotic proteins, SLC7A11 and GPX4, was also observed during 3-MA pretreatment (Fig. 6B). These findings suggest that TFP induces autophagy-mediated ferroptosis in oral cancer cells.

Moreover, pretreatment with the antioxidant NAC attenuated the TFP-induced elevation in intracellular Fe^2+^ and MDA levels (Fig. 6C). The reduced expression of the pro-ferroptotic proteins, NCOA4 and FTH1, and the increased expression of the anti-ferroptotic proteins, SLC7A11 and GPX4, were also observed during NAC pretreatment (Fig. 6D). These results indicate that TFP induces autophagy-mediated ferroptosis associated with ROS in oral cancer cells.

### TFP induces ferroptosis in OSCC cells by targeting GPX4

**Figure 7:**
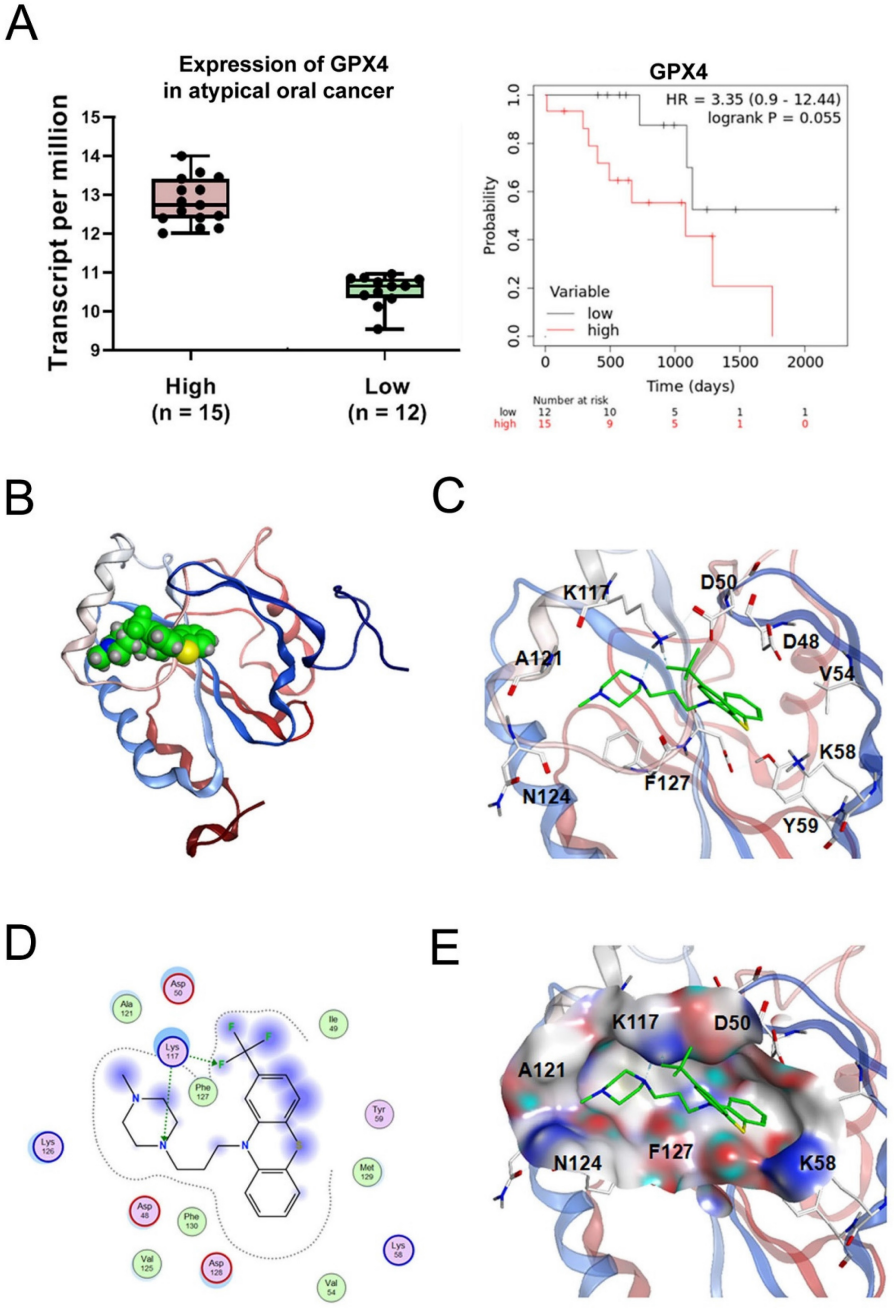
** Interaction pattern between TFP and human GPX4. (A)** The expression of GPX4 in oral cancer was analyzed using the GEPIA database. Relationship between the expression of GPX4 and the overall survival of oral cancer patients was analyzed using the Kaplan-Meier Plotter database. **(B)** A GPX4/TFP complex. GPX4 is shown as a red and blue ribbon, and TFP is green. **(C)** TFP binding area and the contact amino acids (several amino acids were hidden for display convenience). **(D)** The 2D binding mode of GPX4/TFP complex, with a docking score of -6.07 kcal/mol. **(E)** The molecular surface of the interaction region (red is the negatively charged region, blue is the positively charged region, and gray is the hydrophobic region). Amino acid K117 forms hydrogen bonds with TFP.

GPX4, a key regulator of ferroptosis, has higher expression levels in tumor tissues compared with normal tissues, with the higher GPX4 expression levels associated with shorter overall survival (Fig. 7A). To explore the most likely target protein of TFP in the progression of ferroptosis, we used MOE software to make predictions. According to the results, one possible target of TFP was identified as GPX4. Further analysis of the optimal mode between TFP and GPX4 was studied (Fig. 7B). Molecular surface analysis showed that the basal area of GPX4 rendered hydrophobic interactions to the compounds and the surrounding residues of GPX4 were negatively or positively charged for hydrogen bond interactions, such as residue K117, which formed hydrogen bonds with the nitrogen/fluoride atoms of TFP (Fig. 7C). Other charged residues, such as D48, D50, K58, K126, and D128, with several hydrophobic or non-polar amino acids, such as V125, F127, M129, and F130, surrounded the TFP (Fig. 7D, E). Based on these results, we propose that TFP induces ferroptosis in oral cancer cells by targeting GPX4.

### TFP inhibits the growth of oral cancer *in vivo*

**Figure 8:**
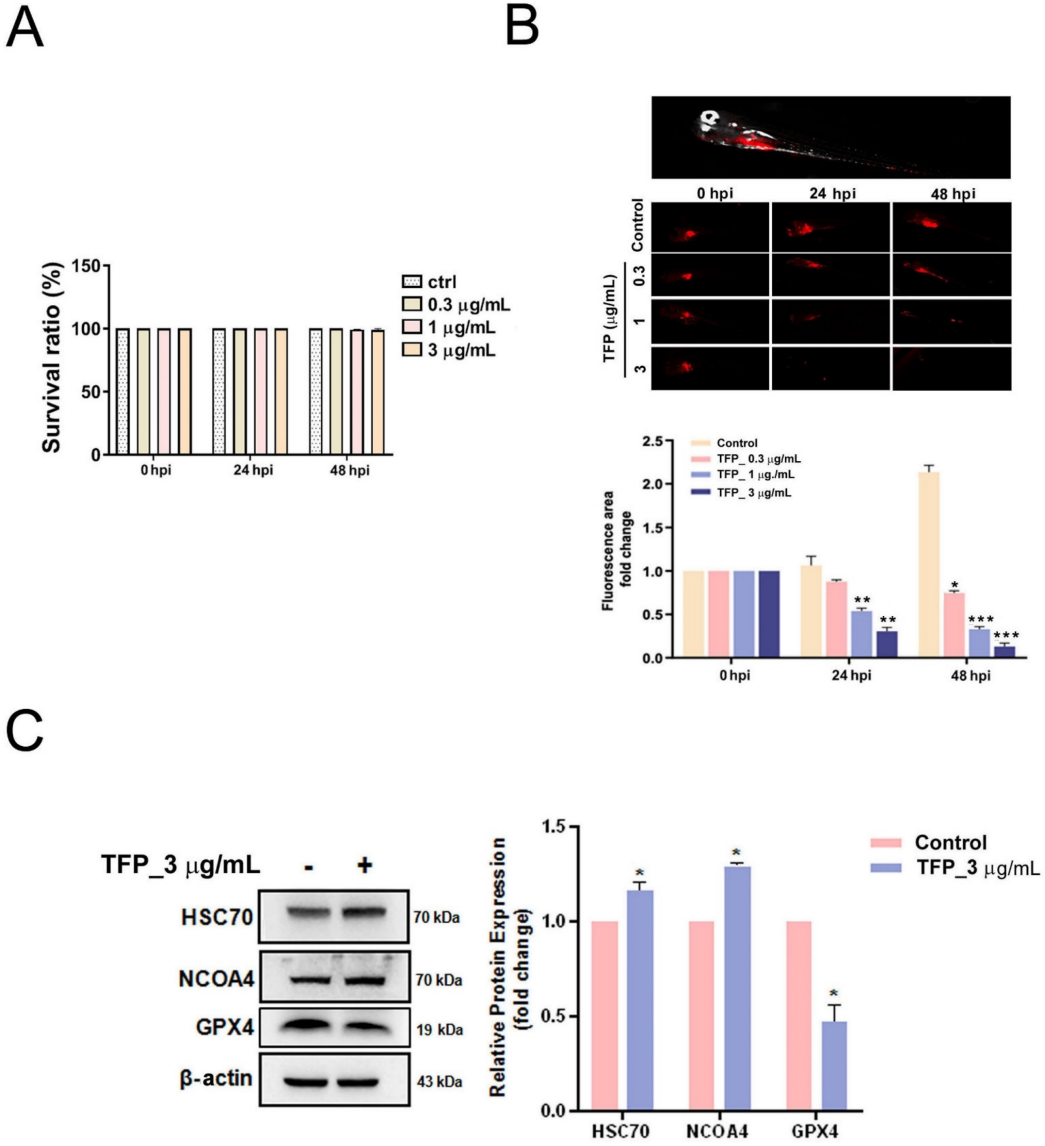
** TFP inhibits tumor growth in a zebrafish xenograft model**. **(A)** TFP at the specified concentrations and time points had no detectable toxicity. **(B)** HSC-3 cells, labeled with the red fluorescent dye CM-DiI, were injected into the yolk sac of zebrafish 48 h post-fertilization. Tumor size was inferred from the intensity of red fluorescence. Zebrafish xenografts were treated with the indicated concentration of TFP and observed at 24 and 48 h post-injection (hpi). The proliferation of tumor cells with or without TFP treatment was quantitatively analyzed. Data are reported as means ± SD (n≥3). Statistical significance is indicated as follows: *P < 0.05, **P < 0.01, ***P < 0.001 compared to the control. **(C)** Western blot analysis was performed to assess the expression of ferroptosis-related proteins, with β-actin serving as the loading control. The histogram illustrates the statistical analysis of the relative expression levels of these proteins. Data are shown as means ± SD (n=3). Statistical significance compared to the control is indicated as *P < 0.01.

Finally, we established a zebrafish xenotransplantation model with Dil-labeled HSC-3 oral cancer cells to monitor oral cancer growth and its response to TFP. Zebrafish at 72 h after fertilization were randomly divided into four groups, and were treated with vehicle (control group) or with TFP (0.3, 1 or 3 μg/mL). The results demonstrated that TFP exerted stronger inhibitory effects on overall tumor growth than the control group after 24 and 48 h post-injection (hpi) (Fig. 8B). In addition, TFP did not lead to developmental toxicity at the indicated concentrations (Fig. 8A). Next, we examined whether TFP affected the GPX4-mediated ferroptosis pathway. We found that pretreatment with TFP (3 μg/mL) suppressed the expression of GPX4, but increased the expression of HSC70 and NCOA4 in the HSC-3-induced zebrafish xenotransplantation model (Fig. 8C). These results suggest that TFP inhibits the growth of oral cancer *in vivo*.

## Discussion

Ferroptosis, a distinct form of cell death driven by iron-dependent lipid peroxidation, offers promising therapeutic potential for various types of cancers 26, 27. In this study, we examined the impact of TFP, an FDA-approved antipsychotic drug, on oral cancer cells. Our study identified the mechanism by which TFP exerts its potential anti-tumor effects, specifically through the induction of ROS, which results in autophagy-mediated ferroptosis.

Intracellular ROS are produced primarily in mitochondria, as well as in peroxisomes and the endoplasmic reticulum, and by various metabolic enzymes 28, 29. The generation of excess ROS can cause cell apoptosis, autophagy, lipid peroxidation, and DNA damage 30, 31. Ferroptosis is a type of regulated cell death that relies on iron-catalyzed lipid peroxidation, largely driven by ROS 32. In this pathway, ROS facilitate the peroxidation of lipids in cell membranes, leading to the loss of membrane integrity and ultimately causing cell death 31. Our findings indicate that TFP significantly increases lipid-derived ROS levels in oral cancer cells. Additionally, we demonstrated that TFP induces mitochondrial damage in oral cancer cells. Mitochondria play a crucial role in energy production and the regulation of apoptotic pathways. When mitochondrial function is impaired, it can lead to the release of pro-apoptotic factors, which further contribute to oxidative stress and facilitate the occurrence of ferroptosis 33. The combined effects of ROS accumulation and mitochondrial damage by TFP foster a cellular environment that is favorable for ferroptosis.

Autophagy, a cellular degradation and recycling process, can act as both a survival and death mechanism in cancer 34. Accumulating studies have revealed the crosstalk between autophagy and ferroptosis. Recently, ferroptosis has been described as an autophagic cell death process, and autophagy is essential to ferroptosis by regulating iron homeostasis and ROS generation 35, 36. A major intracellular iron storage protein is ferritin. A reactive iron (Fe^2+^) produces toxic Fenton-type oxidative reactions, whereas ferritin stores a less toxic state of iron (Fe^3+^) 37. In addition, ferritinophagy is the process of releasing chelated iron from ferritin due to autophagy promoting ferritin degradation 38. The present study suggests that TFP-induced autophagy may promote ferroptosis, potentially through the degradation of ferritin, which leads to the accumulation of free iron. The increased levels of free iron can then catalyze lipid peroxidation, ultimately triggering ferroptotic cell death.

The SLC7A11/GPX4 axis plays a crucial role in the regulation of ferroptosis 39. A cystine/glutamate antiporter system known as Xc- contains SLC7A11, which facilitates the exchange of cystine for glutamate during cystine import 40 that is essential for the synthesis of glutathione (GSH), a crucial antioxidant compound in the body 41. The GSH-reducing enzyme GPX4 makes use of the reduction of lipid hydroperoxides to non-toxic lipid alcohols, which prevents the oxidation and ferroptosis of lipids 42. The results of our study demonstrate that TFP inhibits the activity of GPX4, which was confirmed by a molecular docking analysis. Subsequently, the accumulation of lipid peroxide triggered the induction of ferroptosis. Meanwhile, the present study provides evidence that the expression levels of GPX4 in oral cancer biopsy samples were inversely correlated with patient prognosis. Therefore, the anticancer activity of TFP to selectively target GPX4 highlights its therapeutic potential in the treatment of oral cancer. Several studies demonstrated other pathways also play roles in regulating ferroptosis including the mTOR pathways (43, 44). Further research is needed to determine their potential roles in that process.

Cancer cells typically exhibit elevated levels of glycolytic activity, which result in an increase in ATP production to meet their high energy demands 17, 18, 45, 46. The present study demonstrated that TFP reduces both glycolysis and oxidative phosphorylation (OXPHOS) activity in OSCC cells, as well as basal respiration rates. Therefore, TFP significantly decreases mitochondrial respiration. Apoptosis-related pathways are inhibited by various changes in mitochondrial activity and functionality in cancer cells as a result of decreased MMP caused by apoptosis-inducing factors (e.g., cytochrome C) 45. Based on our results, TFP decreases the MMP in OSCC cells, suggesting that it induces mitochondrial dysfunction, causing the cells to undergo apoptosis.

In conclusion, this study provides evidence that TFP induces ferroptosis in oral cancer cells through a multifaceted mechanism that includes ROS elevation, autophagy, the inhibition of SLC7A11/GPX4 signaling and mitochondrial damage in order to achieve ferroptosis in oral cancer cells. These findings contribute to our understanding of the anti-cancer effects of TFP and highlight the induction of ferroptosis as a possible therapeutic strategy in the treatment of cancer.

## Figures and Tables

**Figure 9:**
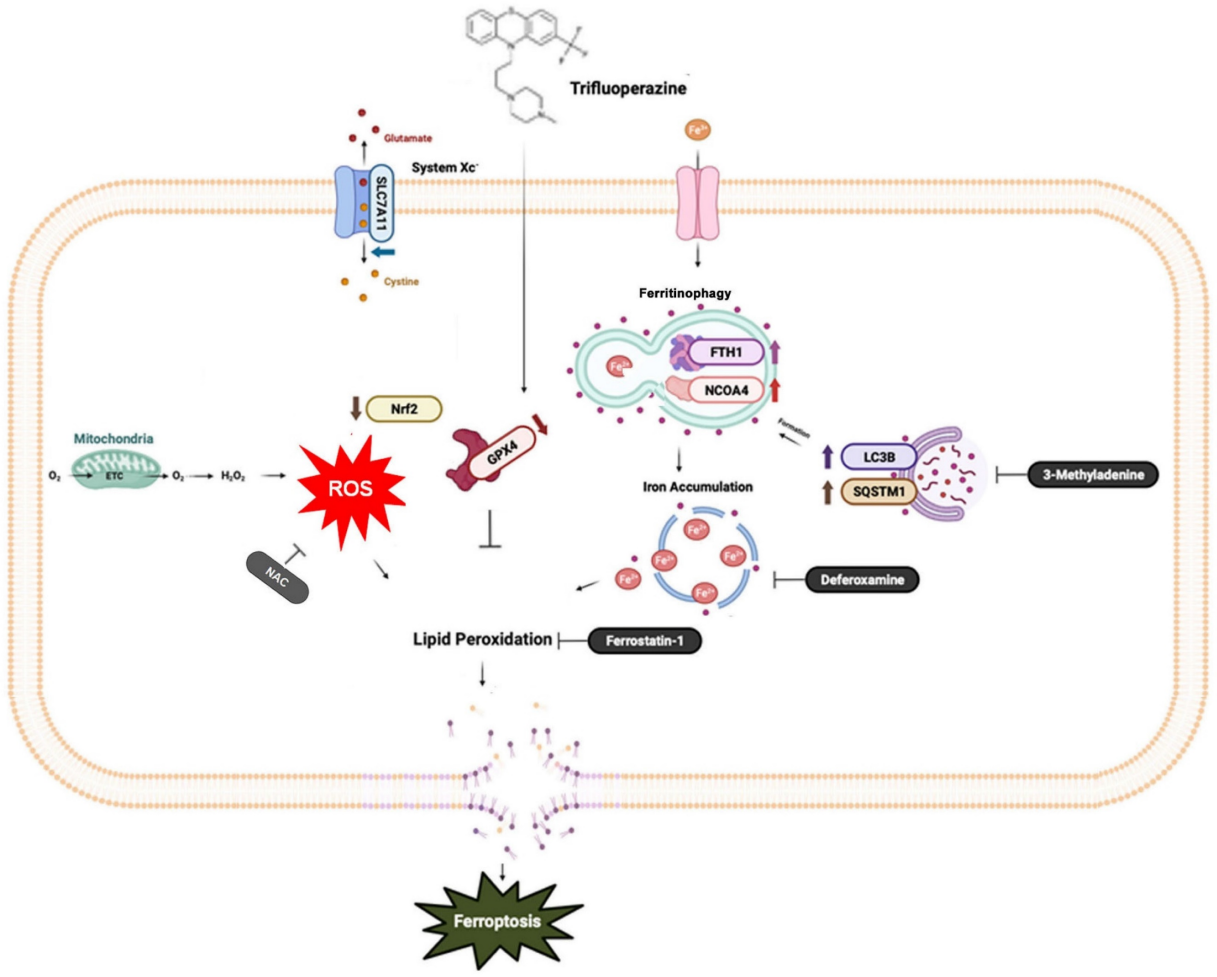
** A diagrammatic illustration of the proposed molecular mechanism responsible for the anti-oral cancer effects of TFP.** TFP induces SLC7A11/GPX4-mediated ferroptosis through the ROS/autophagy pathways.
